# Comparison of Malocclusion Prevalence, Type and Severity between Cerebral Palsy and Healthy Subjects: A Prospective Case-Control Study

**DOI:** 10.3390/jcm11133711

**Published:** 2022-06-27

**Authors:** Victoria Martínez-Mihi, Vanessa Paredes-Gallardo, Francisco-Javier Silvestre, Javier Silvestre-Rangil

**Affiliations:** 1 Department of Stomatology, Valencia University Medical and Dental School, C/Gascó Oliag 1, 46010 Valencia, Spain; vmmihi@gmail.com (V.M.-M.); vanessa.paredes@uv.es (V.P.-G.); francisco.silvestre@uv.es (F.-J.S.); 2 Unit of Stomatology, Doctor Peset University Hospital—FISABIO, Avda. Gaspar Aguilar 90, 46017 Valencia, Spain

**Keywords:** cerebral palsy, malocclusion, dental aesthetic index, DAI

## Abstract

Background: To analyze the prevalence, type and severity of malocclusions in a group of patients with cerebral palsy (CP) using a facial and occlusal analysis and the Dental Aesthetic Index (DAI). Methods: A prospective, case–control study was made of two groups, a cerebral palsy and a control group, with the determination of the facial and occlusion analysis in the three spatial planes. The Dental Aesthetic Index (DAI) was used to assess the severity of malocclusion. Results: The patients with CP presented a higher prevalence of increased facial lower third height and a greater tendency towards right-side canine and molar class II malocclusion, narrower transverse relationship and crossbite. The DAI scores were statistically significantly higher in the CP group. Increased physical impairment in the CP group was associated to greater DAI scores. Conclusions: The prevalence and severity of malocclusion were significantly greater in the CP group. The type of malocclusion predominantly found in these patients was molar class II, with open bite, increased overjet and a narrow arch. The CP group also presented mixed breathing with higher DAI scores and decreased facial lower third height.

## 1. Introduction

Cerebral palsy (CP) comprises a group of body posture and motility disorders that limit patient activity and are the consequence of developmental alterations of the brain during the fetal or nursing infant stages of life [1]. The prevalence of CP ranges from 1.2–3.6 cases per 1000 live births [2]. The diagnosis of CP is based on the clinical features [3,4] and there is no curative treatment for the disease [5].

With regard to the oral manifestations of CP, malocclusion has been the subject of study due to its functional, aesthetic, psychological and social impact [6,7]. At present, there is widespread agreement that the development of the craniofacial structures and of the final position of the teeth is the result of the interaction of genetic and environmental factors [8,9]. In terms of the latter, the strength generated by the muscles of the orofacial region both at rest and during function can influence the development of malocclusion through muscle feedback mechanisms and soft tissue stretching effects [6,8,9,10]. While there is abundant information in the literature on malocclusions in the general population, the data referred to individuals with CP are more limited. Due to the mainly physical differences in the environmental conditions under which malocclusion develops in these patients, the prevalence of malocclusion could be expected to be different and presumably higher [11,12].

In recent years, some articles have appeared in the literature on dental malocclusions among patients with CP [13,14,15,16], but only some have used the Dental Aesthetic Index (DAI) to assess the prevalence, type and severity of these malocclusions in such individuals [7,17,18]. Although there are some studies that assess respiratory pathology in patients with CP [15,19], there are few that relate the type of breathing with the development of malocclusion [20].

Therefore, the aim of the present study was to analyze the prevalence, type and severity of malocclusions in a group of patients with CP and to establish comparisons with a healthy control group without physical or mental disabilities using a facial and occlusal analysis and the Dental Aesthetic Index (DAI).

## 2. Materials and Methods

Power analysis showed that a sample size of 120 patients would provide a 78% probability of detecting a statistically significant medium effect (f = 0.25) between the two groups of individuals using an ANOVA model at a confidence level of 95%.

### 2.1. Study Design and Sample

A prospective, observational cross-sectional case–control study was carried out in accordance with the ethical guidelines of the Declaration of Helsinki referred to medical research in human subjects and was approved by the Clinical Research Ethics Committee of Dr. Peset University Hospital, Valencia, Spain (procedural registration # CEIC CODE 42/14). All patients’ parents or tutors were informed of the study design beforehand, and informed consent was given prior to participation. Six patients did not wish to participate for personal reasons.

The study data were comprised of extraoral facial analysis (facial thirds measurements), intraoral occlusal analysis (sagittal, vertical and transverse plane), Dental Aesthetic Index (DAI) analysis, diet and oral habits questionnaire and breathing-type study of the patients with CP and of the control group.

The study population consisted of males and females with CP from two special education centers and a dental hospital in Valencia (Spain), while the control group consisted of healthy individuals of either gender with no physical or mental disabilities, either parents or tutors of the CP patients or patients who attended the hospital to have their wisdom teeth removed for any reason.

The inclusion criteria were individuals aged over 12 years; absence of previous orthodontic treatment; no missing permanent first molars; no more than two missing teeth in the upper and lower anterior sectors. The study group was moreover required to present CP independently of the degree of severity, cooperation or associated disease conditions. Meanwhile, the exclusion criteria were cases with a doubtful diagnosis of CP and patients failing to cooperate in the study evaluations.

Randomization was performed using an “echo $ [RANDOM%2 + 1]” command in the Mac OSX operating system, which generates random numbers allowing a value of 1 or 2 to be assigned to each patient. The subjects assigned as “1” were included in the study, while the rest were excluded to the effects of analysis.

### 2.2. Determinations of the Type of Cerebral Palsy (CP)

Classification of the study subjects according to the diagnosis of the type of CP and the type of mental disability was based on the health records defining the degree of patient disability. The following types of CP were distinguished: spastic with its different manifestations, tetraplegia, diplegia and hemiplegia, and athetotic, ataxic and mixed forms. The degree of mental disability in turn was classified as mild, moderate, severe or profound. The severity of the physical alterations of CP was assessed based on the criteria of Papazian [13], distinguishing among mild, moderate and severe presentations.

### 2.3. Extraoral Facial Thirds Measurements

**Figure 1:**
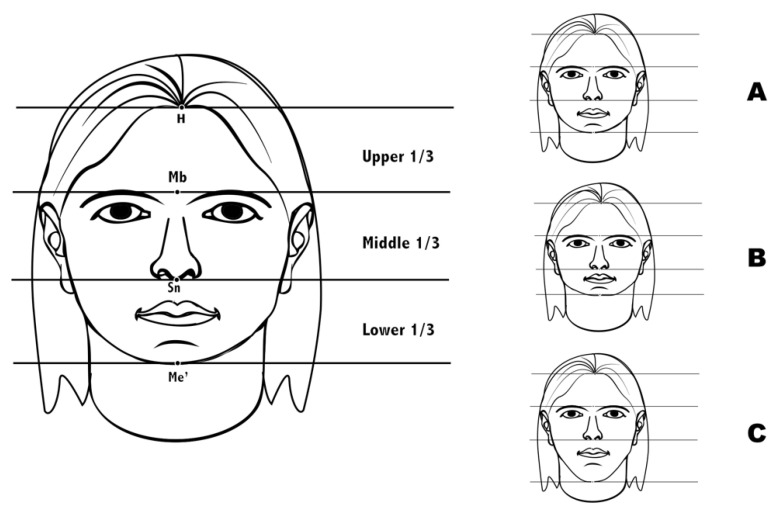
Facial thirds. Upper third (hairline to midbrow), middle third (midbrow to subnasale) and lower third (subnasale to soft tissue menton). (**A**) = equal facial thirds, (**B**) = shorter lower third, (**C**) = longer lower third.

The facial thirds measurements were determined during the clinical examination of patients using a blunt Spitz^®^ compass, measuring the distance (mm) between them as Arnett’s study [21] as Figure 1 shows. A balanced face was taken to represent equal facial thirds (Figure 1A), while a short length was defined by a shorter lower third (Figure 1B), and large length was defined by a longer lower third (Figure 1C).

### 2.4. Intraoral Occlusal Measurements 

**Figure 2:**
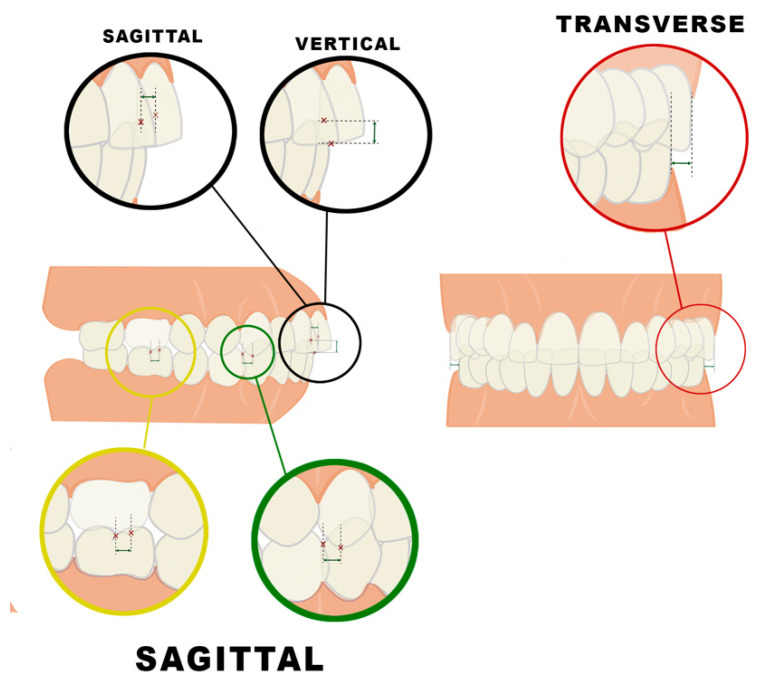
Intraoral occlusal measurements.

Occlusion was analyzed in the three spatial planes (Figure 2 and Table 1). In the sagittal plane, measurements were made for the angle molar and canine class [22] and overjet (mm). In the vertical plane, overbite was also measured (mm). When overbite was −1 mm or below, these patients were classified as open bite patients for both the CP and the control group. Finally, the transverse relation between the buccal surfaces of the upper and lower posterior molars was measured. A CP15 periodontal probe (Hu-Friedy, Chicago, IL, USA) was used to record the distances.

### 2.5. Dental Aesthetic Index (DAI) Components

The Dental Aesthetic Index (DAI) was used to assess the diagnosis and severity of malocclusion [14] as Table 2 shows. The ten DAI components were measured directly in the oral cavity with the same CP15 periodontal probe. The subjects were classified according to DAI scores in groups 1, 2, 3 and 4.

### 2.6. Diet and Oral Habits Questionary

A questionnaire was given to the parents or tutors of the CP patients about the type of diet they were consuming at the time of the study (solid, shredded, semi-solid and or fed by tube) and the presence of any bad oral habits (pacifier, finger/objects sucking or others). Control group individuals were asked about these issues as well.

### 2.7. Breathing Type

The breathing types of both groups was also explored using the modified Glatzel mirror test and classifying it as nasal, oral or mixed.

### 2.8. Statistical Analysis

The reliability and validity of the results was ensured through double assessment of the whole study sample. Within-examiner error was estimated using the Dahlberg method and the coefficient of variation (CV) for the assessment of continuous variables and the kappa concordance index for categorical parameters.

The data obtained were entered on a spreadsheet using Microsoft Excel 2011 (Microsoft Corp, Redmond, WA, USA) and transferred to the statistical software package SPSS v. 22.0 (IBM, Endicott, NY, USA) for analysis.

Bivariate analysis was performed using the chi-squared test (χ²), Student’s *t*-test and the Kruskal–Wallis test. Multivariate analysis in turn was performed to explore relationships between a response and a factor, with assessment of whether the relationship was conditioned by the group of individuals, the control group or the study. Analyses of variance (ANOVA) and covariance (ANCOVA) were used for this purpose.

## 3. Results

### 3.1. Reproducibility

To calculate intra-examiner reproducibility, the same observer (VMM) repeated all measurements within a one-month period between the first and second measurements, the mean differences between the first and second measurements were seen to be small, not exceeding 0.5 units for all the variables. Reproducibility was therefore quite good for all the measurements and proved to be very high for the DAI score, with a coefficient of variation (CV) of 0.8% and Dahlberg d of 0.27. Concordance of the categorical variables was seen to be good to very good (kappa index = 1).

### 3.2. Sample Size and Groups

Participants were selected from a total of 180 patients after applying the inclusion and exclusion criteria detailed above, the final sample consisted of 120 patients divided into two groups (60 in the CP group and 60 in the control group) with a mean age of 33.3 ± 10.7 years. Regarding the effect of the patients’ sex on the results, it was confirmed that there were no significant differences in the different associations and correlations evaluated. In the CP group, the prevalence of malocclusion was 96.7% in women and 93.3% in men, without significant differences (*p* = 1.000, Fisher’s exact test). Both results were also significantly higher than those registered among the respective control group (*p* < 0.001). Therefore, the results are presented together for both sexes without distinction.

The final sample was comprised of the following:

The mean age in the CP group was 32.1 ± 10.8 years (range 12–58), versus 34.6 ± 10.5 years (range 20–59) among the control group. The total study sample consisted of 68 females (56.7%) and 52 males (43.3%). The gender distribution was 30 females (50%) and 30 males (50%) in the CP group versus 38 females (63.3%) and 22 males in the control group (36.7%).

In relation to the type of CP, 34 patients suffered tetraplegia (55.6%), three diplegia (4.9%), two hemiplegia (3.2%), 8 athetoid CP (13%), four ataxic CP, and 9 mixed CP (14.7%). The most frequent presentations were spastic CP with severe physical impairment and moderate mental disability. The affectation degree was severe for 58% of the total, with severe–profound mental retardation for 33.3%. The degree of malocclusion increased significantly according to the degree of CP (*p* = 0.027) and, with a strong trend, according to the extent of mental retardation (*p* = 0.063). No relevant association was found between the degree of malocclusion and the type of CP. A total of 73.3% of the CP individuals were having any special medication.

### 3.3. Extraoral Facial Thirds Measurements

The results of the the relations of facial thirds measurements showed that in the CP group, 15% (9 patients) presented equal thirds, 6.7% (4 patients) decreased lower third while the great majority, 78.3% (47 patients) had increased lower third being the difference statically significant. On the contrary, the majority of the control group, 75% (45 patients) presented equal thirds, 15% (9 patients) increased lower third and 10% (6 patients) decreased lower third being the difference statically significant again.

### 3.4. Intraoral Occlusal Measurements

Malocclusion prevalence in the CP group was 95% (CI 95% 86.1–98.9%), significantly higher than the prevalence of 26.7% of the control group (*p* < 0.001).

Occlusal measurements of both groups are shown in Table 3. Patients with CP showed in the sagittal plane a greater tendency towards right molar and canine class II, and increased overjet and decreased overbite (open bite) in the vertical plane; finally, a greater narrowness of the maxilla and posterior crossbite was also found.

### 3.5. Dental Aesthetic Index (DAI) Components

The results referring to the DAI scores and their relationship between the two groups are reported in Table 4. The DAI scores between the groups proved statistically significant difference being the CP scores much higher (*p* < 0.001). Two thirds of all CP patients presented a ‘very severe’ malocclusion based on the value of the DAI score.

The data referring to the relationships between the DAI score and different intraoral parameters are shown in Table 5. Statistically significant correlations were observed between the DAI score and increased overjet, the presence of an open bite, transverse narrowness, and increased interincisal diastema distance. Furthermore, the vertical relationship showed an increase in DAI score depending upon whether the subject suffered from CP or not—a clear association being established with the presence of CP.

The analysis of the relationship between the DAI scores and the diagnostic factors showed no correlation in terms of the type of CP, though an association was observed in relation to the degree of physical impairment—the greater the latter, the higher the DAI score. In relation to intellectual disability, although statistical significance was not reached, there was a clear tendency towards an association between them. The degree of malocclusion was seen to increase in the groups with severe and profound intellectual disability.

### 3.6. Diet and Oral Habits Questionary

Referring to the type of diet, 45% of the CP patients were eating a solid diet at the time of the study, 30% a shredded diet, and 18.3% a semi-solid diet, while 6.7% were fed by tube. All of the patients in the control group ate a solid diet. The DAI score determined a direct relationship between the most severe malocclusion and patients who were feed by tube. Patients with a solid diet had a score of 37.5 ±13.7, with a semi-solid diet had a score of 43.3 ± 12.2, with a shredded diet a score of 64.7 ± 34.4, and lastly, patients fed by tube had a score of 88.3 ± 34.1.

With respect to the presence of any bad oral habits, the frequency was very low since just 4 patients out of 60 presented any of them in the CP group while none of the patients in the control group presented any of them.

### 3.7. Breathing Type

Regarding the breathing type, 93.3% of the control group presented nasal breathing compared to 23.3% of the CP, while the majority of the CP group presented mixed breathing, 76.7% compared to 6.7% of the control group, with the differences being statistically significant. No patient in either group presented as exclusively mouth breathing. Only 4 subjects from the control group presented mixed respiration, so the analysis is just limited to CP patients, evaluating the relationship between the DAI scores and the type of breathing. The differences are statistically significant (*p* = 0.007), with higher DAI scores in mixed breathing. Fourteen CP patients presented nasal breathing with a DAI score of 34.5 SD 8.5 (min. 23.0–max. 49.0, and median 32.0) while forty-six CP patients had mixed breathing with a DAI score of 54.8 SD 29.5 (min. 22.0–max. 133.0, and median 44.5). The relationship between breathing type and diet was also significant. The percentage of patients with mixed breathing rises as the patient has more difficulty chewing and the food is more crushed (Table 6).

Likewise, facial thirds measurements are also related to the breathing type that the patient presents. The results showed that in the mixed group, 44.4% (4 patients) presented equal thirds, 25% (1 patient) decreased lower third, while the great majority, 87.2% (41 patients), had increased lower third with the difference being statically significant. On the contrary, in the nasal group, 55.6% (5 patients) presented equal thirds, the majority of the group, 75% (6 patients), decreased lower thirds, and 12.8% (3 patients), increased lower thirds with the difference being statically significant again.

Finally, a complete summary of statistical tests was carried out to compare all the variables. The differences were very numerous, statistically significant for the majority of them. Epilepsy and medication were common among CP patients (<0.001 *** Chi^2^). DAI score was significantly higher in CP patients (<0.001 *** Student’s *t*-test). Most of the patients presented an increased lower facial third (78.3%) compared to 15% of the controls (<0.001 *** Chi^2^). Breathing type was affected in CP patients, as they do not present nasal respiration (<0.001 *** Chi^2^). Molar class parameters were significantly higher in CP patients (0.039 *, Student’s *t*-test), as was the overjet (<0.001 *** Student’s *t*-test) while vertical parameters were reduced in these patients (<0.001 *** Student’s *t*-test). Three-quarters of CP patients presented some alteration of the transverse relation (<0.001 ***Chi^2^). 56.7% of CP patients presented separation (<0.001 *** Chi^2^), also with a significantly higher mean diastema (0.001 **, Student’s *t*-test). Only the maximum irregularity in the jaw differed in both groups, being higher in CP patients (<0.001 *** Student’s *t*-test).

## 4. Discussion

The aim of the present study was to analyze the prevalence, type and severity of malocclusions in a group of patients with CP using facial and occlusal measurements and the DAI, since only few studies were found in the literature on this topic [7,17,18].

The present study had a large sample size of 120 patients compared with other studies, and just one other study had a control group [7]. Intra-examiner error was low for all the measurements and so reproducibility was high.

For the inclusion criteria, no more than two missing teeth in the upper and lower anterior sectors, independent of the type of replacement prosthesis used, were accepted, as this would impede an adequate assessment of occlusion. The sample included patients with an age range of between 12–59 years old; the mean age was 33.3 years, so it should be noted that malocclusion may vary for various reasons, such as periodontal health or occlusal trauma, in middle-aged adults compared to children.

The present study suffered certain limitations. First, even though clinical examination is always the best way to determine angle class classification, this is sometimes influenced by skeletal discrepancy (Skeletal Class I/II/III). Taking additional lateral radiographs would have been a good option in order to analyze this influence, but this is sometimes a difficult process in CP patients since they cannot keep their heads straight and, in addition, it would have implied taking a radiograph not necessary for this study. Second, the DAI was measured directly in the oral cavity being difficult to do it especially in CP individuals. This was done to avoid scanning the mouth of these patients or even taking impressions.

In our study, the percentage of CP patients with severe malocclusion was seen to be similar to that reported by other authors [7,17,18], although the literature only reports one other study with a control group, where a clear association was likewise observed between CP and the DAI score [7], thus evidencing that patients with CP suffer more severe malocclusions than the general population.

With regard to the type of CP, no association to malocclusion was observed in our study, in contrast to other authors that have reported a correlation mainly to spastic CP [12,23,24,25]. There may be two explanations for this situation. On one hand, we found spastic CP to be the most common clinical presentation of the disease, and on the other, as reported by Foster et al. [26], this presentation can exhibit alterations in the size of the skull and facial bones. In contrast, a relationship between the degree of intellectual disability and the severity of CP and the DAI score was observed by us. Specifically, severe physical and mental problems were associated with higher DAI scores, i.e., to more severe malocclusion, thus showing increased general disease involvement to be associated with greater alterations to craniofacial development of the patient.

With regard sagittal relation, to the molar relationship, and as evidenced by the DAI scores, the CP group was characterized by a greater presence of individuals with abnormal relations compared to the control group. In agreement with other authors, the analysis based on the Angle classification [18] showed class III to be the least prevalent malocclusion in both groups of individuals, while class I was found to be the most prevalent presentation among the control group [7,12,17,18,21,22,23,24,25,27,28,29,30,31,32]. In coincidence with other investigators [21,22], an association between molar and canine class II and CP was observed in our study, although other authors have observed no such association [12,28]. Such variability with our results may be due to the fact that these studies included patients with temporary, mixed and permanent dentition. On considering overjet and independently of the index used, most studies in patients with CP have reported higher values than among the control population [7,12,17,23,24,28,30,31,32], in coincidence with our results. Furthermore, in line with other studies, a significant association between overjet and the DAI score was found, thereby showing it to be correlated to the severity of malocclusion [11,12,23,24,27,31].

On considering the vertical relation and in concordance with most data reported in the literature, the frequency of open bite among CP patients was found to be greater than in the control group, although the mean values in our study were comparatively greater than those published by others [7,12,23,28,32]. This tendency towards increased open bite has been associated by some investigators to the type of CP, since the occlusal features of spastic CP have been shown to differ from those of the other presentations of the disease [24]. According to some authors, this situation can be explained by a worsening of malocclusion with age [18,23,25,27,28,31]. These two arguments would clarify why we recorded higher values, since spastic CP predominated in our series, and the patients were also older than in other studies.

With regard to the transverse relation, the patients with CP examined in our study presented a high and significant incidence of narrow arches. Only one study [12] in the literature has found similar results. This issue may be explained in terms of a lack of muscle tone in CP patients and the type of diet, since 55% of the CP patients in our study were not consuming a solid diet. Bad oral habits had no influence in this study due to their low prevalence in CP patients.

On analysis, the prevalence of upper and lower incisor crowding and spacing was seen to be greater among the CP group. As suggested by other authors [7,18,27], these characteristics are related to CP but not to the DAI score. As a result, they are seen to be altered in patients with CP but show no correlation to the severity of the disease. Interincisal diastemas have been found to be more common in patients with CP than in the healthy population [7,18,28], and in this study a significant association to both the DAI score and CP was observed, in agreement with the results of other authors [10].

The type of malocclusion predominantly found in the patients with CP was molar class II, with open bite, increased overjet and a narrow arch. In this regard, the muscle disorders inherent to CP might explain why malocclusion is more severe in these individuals. Such alterations in muscle control would affect the orofacial muscles and ultimately conditioning the final position of the teeth.

Due to the low frequency of any bad oral habits in both groups, no significant results were found in this study. The age of the sample of our study has to do with the absence of bad oral habits being more frequent at younger ages.

In our patients, we found a relationship between mixed breathing and DAI; other authors have also previously found a relationship between breathing and malocclusion (20). However, they found a relationship with oral breathing and we with mixed breathing, which could be due to the fact that in our sample we did not find patients with exclusive oral breathing.

The orthodontic treatment of subjects with CP is limited mainly by the lack of patient cooperation, most of these individuals being candidates for orthognathic surgery. These are patients with a great need for orthodontic treatment that often proves very complex, with aesthetic and functional alterations—so early diagnosis and treatment should be done in an attempt to prevent major occlusal disorders worsening with age.

## 5. Conclusions

It can be concluded that the prevalence and severity of malocclusion were significantly greater in the CP group. The type of malocclusion predominantly found in these patients was molar class II, with open bite, increased overjet and a narrow arch. The CP group also presented mixed breathing with higher DAI scores and a decreased facial lower third.

## Figures and Tables

**Table 1:** Intraoral occlusal measurements in the three spatial planes; sagittal, vertical and transverse.

SAGITTAL PLANE			
**MOLAR CLASS** Distance between the mesiobuccal cusp of the upper first molar and the mesiobuccal sulcus of the lower first molar	I	II	III
	=0 mm	>0 mm	<0 mm
**CANINE CLASS** Distance between the cusp of the upper canine and the contact point between canine and lower first premolar	I	II	III
	=0 mm	>0 mm	<0 mm
**OVERJET** Distance between the labial surface of the lower central incisor and the incisal margin of the upper central incisor considering normal 0–4 mm			
**VERTICAL PLANE**			
**OVERBITE** Distance between upper and lower incisal margins. Considering normal 0–4 mm. −1 mm or below was considered open bite			
**TRANSVERSE PLANE**			
**TRASNVERSE RELATION** Distance between the buccal surfaces of the upper and lower posterior molars (unilateral or bilateral nature)	NORMAL	NARROW	CROSSBITE
	=2 mm	Narrow = 0–2 mm	Crossbite < 0 mm

**Table 2:** Dental Aesthetic Index (DAI) 10 components. Groups by final score.

DAI Components		SCORE		
		0	1	2
1	Number of visible missing teeth in both arches (incisors, canines and premolars)			
2	Assessment crowding in the incisal segments	No crowded segments	One crowded segment	Two crowded segments
3	Assessment spacing in the incisal segments	No spaced segments	One spaced segment	Two spaced segments
4	Measurement of any midline diastema (mm)			
5	Largest anterior irregularity on the maxilla (mm)			
6	Largest anterior irregularity on the mandible (mm)			
7	Measurement of anterior maxillary overjet (mm)			
8	Measurement of anterior mandibular overjet (mm)			
9	Measurement of vertical anterior open bite (mm)			
10	Assessment of sagittal molar relation; largest deviation from normal either left or right	Normal	1/2 cusp either mesial or distal	One full cusp or more either mesial or distal
	**TOTAL**			
**RESULTS** Group 1 < 25 points = no anomalies with no need of orthodontic treatment Group 2 = 26–30 points, indicative of malocclusion, with optional treatment Group 3 = 31–35, indicative of severe malocclusion, with highly recommendable treatment Group 4 > 36 points, indicative of very severe malocclusion, in which treatment proves crucial				

**Table 3:** Relationship of the intraoral occlusal measurements in the three spatial planes—sagittal, vertical and transverse—between the two groups: Cerebral Palsy (CP) and Control Group. χ²: chi-squared test; t: Student’s *t*-test; *p* < 0.05 *; *p* < 0.001 ***.

VARIABLES	CP Group		Control Group		*p*-Value
	*n*	%	*n*	%	
**SAGITTAL RELATION. RIGHT MOLAR CLASS**					
Class I	26	43.3	41	68.3	0.039 * χ²
Class II	24	40.0	6	10.0	
Class III	9	15.0	13	21.6	
**SAGITTAL RELATION. LEFT MOLAR CLASS**					
Class I	16	26.6	36	60.0	0.194 χ²
Class II	24	40.0	9	15.0	
Class III	18	30.0	15	25.0	
**SAGITTAL RELATION. RIGHT CANINE CLASS**					
Class I	22	36.6	39	65.0	0.007 * χ²
Class II	28	46.6	15	25.0	
Class III	8	13.3	6	10.0	
**SAGITTAL RELATION. LEFT CANINE CLASS**					
Class I	19	31.6	42	70.0	0.018 * χ²
Class II	28	46.6	12	20.0	
Class III	11	18.3	6	10.0	
**SAGITTAL RELATION. OVERJET**					
	Mean ± SD = 5.6 ± 5.0 mm Max (16 mm) Min (−9 mm)		Mean ± SD = 2.4 ± 1.5 mm Max (6 mm) Min (−1 mm)		<0.001 *** t
**VERTICAL RELATION. OVERBITE**					
	Mean ± SD = −7.5 ± 6.7 mm Max (−1 mm) Min (−20 mm)		Mean ± SD = −2.0 ± 1.2 mm Max (−3 mm) Min (−1 mm)		<0.001 *** t
**TRANSVERSE RELATION**					
Normal	16	26.7	52	86.7	<0.001 *** χ²
Narrowing	33	55.0	5	8.3	
Unilateral Cross Bite	9	15.0	1	1.7	
Bilateral Cross Bite	2	3.3	2	3.3	

**Table 4:** Descriptive scores of the Dental Aesthetic Index (DAI) components and bivariate analysis between the DAI score and the two groups: Cerebral Palsy (CP) and Control Group. Χ²: chi-squared test; t: Student’s *t*-test; *p* < 0.001 ***.

GROUP	DAI SCORES										
	≤25		26–30		31–35		≥36		*p*-Value	Mean ± SD (Min–Max) − Median	*p*-Value
	*n*	%	*n*	%	*n*	%	*n*	%			
CP	3	5.0	10	16.7	7	11.7	40	66.7	<0.001 *** χ²	50.1 ± 27.4 (22.0–133.0) − 40.5	<0.001 t ***
Control	44	73.3	10	16.7	3	5.0	3	5.0		23.3 ± 5.3 (14.0–41.0) − 22.0	

**Table 5:** Descriptive relationship between the DAI components and oral variables and the F-test results of the ANCOVA model for principal effects and the interaction of the relationship between the DAI score, oral variables and group (Cerebral Palsy [CP] and Control); *p* < 0.01 **; *p* < 0.001 ***. SD standard deviation.

DAI/OralVariables	CP Group		Control Group		*p*-Value, Factor	*p*-Value, Group	*p*-Value, Interaction
	*n*	%	*n*	%			
CROWDING incisal segments							
0 segment	21	35.0	17	28.3	0.584	<0.001 ***	0.179
1 segment	24	40.0	28	46.7			
2 segments	15	25.0	15	25			
SEPARATION incisal segments							
0 segment	26	43.3	53	88.3	0.467	<0.001 ***	0.562
1 segment	18	30.0	6	10.0			
2 segments	16	26.7	1	1.7			
TRANSVERSE RELATION					0.006 **	<0.001 ***	0.133
SAGITTAL MOLAR RELATION							
Normal	7	11.7	31	51.7	0.242	<0.001 ***	0.519
Semi-cusp	35	58.3	24	40.0			
Complete cusp	18	30.0	5	8.3			
	Mean ± SD		Mean ± SD				
Right Molar Relation	0.9 ± 3.5		−0.2 ± 1.7		0.140	<0.001 ***	0.530
Left Molar Relation	0.6 ± 3.6		−0.1 ± 1.6		0.422	<0.001 ***	0.887
Maxillary Maximum irregularity	1.1 ± 1.2		0.9 ± 1.2		0.073	<0.001 ***	0.734
Mandibular Maximum irregularity	1.9 ± 1.5		1.0 ± 0.7		0.417	<0.001 ***	0.855
INTERINCISAL DIASTEMA	1.0 ± 1.7		0.2 ± 0.7		<0.001 ***	<0.001 ***	0.220
VERTICAL RELATION	−1.0 ± 7.0		2.6 ± 2.0		<0.001 ***	<0.001 ***	<0.001 ***
VERTICAL ANTERIOR Open bite	−7.5 ± 6.7		−2.0 ± 1.2				
OVERJET	5.6 ± 5.0		2.4 ± 1.5		<0.008 ***	<0.001 ***	0.723
Increased overjet	8.80 ± 2.9		5.14 ± 0.37				

**Table 6:** Relationship between breathing type and diet type.

TYPE OF DIET										
BREATHING TYPE	TOTAL		SOLID		SEMISOLID		SHREDDED		FED BY TUBE	
	*n*	%	*n*	%	*n*	%	*n*	%	*n*	%
TOTAL	60	100	27	100	11	100	18	100	4	100
NASAL	14	23.3	12	44.4	1	9.1	1	5.6	0	0
MIXED	46	76.7	15	55.6	10	90.9	17	94.4	4	100

## Data Availability

The datasets generated and/or analyzed during the current study are available from the corresponding author on reasonable request.

## References

[1] Rethlefsen S.A., Ryan D.D., Kay R.M. (2010). Classification systems in cerebral palsy. Orthop. Clin. N. Am..

[2] Blair E. (2010). Epidemiology of the cerebral palsies. Orthop. Clin. N. Am..

[3] Sankar C., Mundkur N. (2005). Cerebral palsy-definition, classification, etiology and early diagnosis. Indian J. Pediatr..

[4] Dougherty N.J. (2009). A review of cerebral palsy for the oral health professional. Dent. Clin. N. Am..

[5] Krigger K.W. (2006). Cerebral palsy: An overview. Am. Fam. Physician.

[6] Winter K., Baccaglini L., Tomar S. (2008). A review of malocclusion among individuals with mental and physical disabilities. Spec. Care Dent..

[7] Miamoto C.B., Ramos-Jorge M.L., Pereira L.J., Paiva S.M., Pordeus I.A., Marques L.S. (2010). Severity of malocclusion in patients with cerebral palsy: Determinant factors. Am. J. Orthod. Dentofac. Orthop..

[8] Proffit W.R. (1978). Equilibrium theory revisited: Factors influencing position of the teeth. Angle. Orthod..

[9] Harvold E., Chieri G., Vargervik K. (1972). Experiments on the development of dental malocclusions. Am. J. Orthod..

[10] Mamaghani S.M., Bode H., Ehmer U. (2008). Orofacial findings in conjunction with infantile cerebral paralysis in adults of two different age groups-a cross-sectional study. J. Orofac. Orthop..

[11] Franklin D.L., Luther F., Curzon M.E. (1996). The prevalence of malocclusion in children with cerebral palsy. Eur. J. Orthod..

[12] Strodel B.J. (1987). The effects of spastic cerebral palsy on occlusion. ASDC J. Dent. Child..

[13] Papazian O., Alfonso I. (1997). Cerebral palsy theraphy. Rev. Neurol..

[14] Jenny J., Cons N.C. (1996). Establising malocclusion severity levels on the dental aesthetic index (DAI) scale. Aust. Dent. J..

[15] Bensi C., Costacurta M., Docimo R. (2020). Oral health in children with cerebral palsy: A systematic review and meta-analysis. Spec. Care Dent..

[16] Medeiros-Rodrigues-Cardoso A., Duarte-Silva C.R., Nóbrega-Gomes L., Marinho-Davino de Medeiros M., Nascimento-Padilha W.W., Cabral-Cavalcanti A.F., Leite-Cavalcanti A. (2020). Prevalence of Malocclusions and Associated Factors in Brazilian Children and Adolescents with CerebralPalsy: A Multi-Institutional Study. Int. J. Dent..

[17] Martínez-Mihi V., Silvestre F.J., Orellana L., Silvestre-Rangil J. (2014). Resting position of the head and malocclusion in a group of patients with cerebral palsy. J. Clin. Exp. Dent..

[18] Vellappally S., Gardens S.J., Al Kheraif A.A., Krushna M., Babu S., Hashem M., Jacob V., Anil S. (2014). The prevalence of malocclusions and its association with dental caries among 12–21 year-old disabled adolescents. BMC Oral. Health.

[19] Oliveira A.C., Paiva S.M., Martins M.T., Torres C.S., Pordeus I.A. (2011). Prevalence and determinant factors of malocclusion in children with special needs. Eur. J. Orthod..

[20] Bakarcić D., Lajnert V., Maricić B.M., Jokić N.I., Vrancić Z.R., Grzić R., Prpić I. (2015). The Comparison of Malocclusion Prevalence Between Children with Cerebral Palsy and Healthy Children. Coll Antropol..

[21] Arnett G.W., Bergman R.T. (1993). Facial keys to orthodontic diagnosis and treatment planning—Part II. Am. J. Orthod. Dentofac. Orthop..

[22] Angle E. (1899). Classification of maloclussion. Dent. Cosmos.

[23] Santos M.T., Masiero D., Ferreira N., Lorenzetti M.R. (2003). Oral conditions in children with cerebral palsy. J. Dent. Child.

[24] Carmagnani F.G., Gonçalves G.K., Corrêa M.S., dos Santos M.T. (2007). Occlusal characteristics in cerebral palsy patients. J. Dent. Child.

[25] Barrionuevo L., Solís F. (2008). Anomalías dento maxilares y factores asociados en niños con parálisis cerebral. Rev. Child. Pediatr..

[26] Foster T.D., Griffiths M.I., Gordon P.H. (1974). The effects of cerebral palsy on the size and form of the skull. Am. J. Orthod..

[27] Pope J.E.C., Curzon M.E.J. (1991). The dental status of cerebral palsied children. Pediatr. Dent..

[28] Oreland A., Heijbel J., Jagell S. (1987). Malocclusion in physically and/or mentally handicapped children. Swed. Dent. J..

[29] De Carvalho R.B., Mendes R.F., Prado R.R., Moita-Neto J.M. (2011). Oral health and oral motor function in children with cerebral palsy. Spec. Care Dent..

[30] Rosembaum C.H., McDonald R.E., Levitt E.E. (1966). Occlusion of cerebral palsied children. J. Dent. Res..

[31] Schwartz S., Gisel E.G., Clarke D., Haberfellner H. (2003). Association of occlusion with eating efficiency in children with cerebral palsy and moderate eating impairment. J. Dent. Child..

[32] Du R.Y., McGrath C., Yiu C.K.T., King N.M. (2010). Oral health in preschool children with cerebral palsy: A case-control community-based study. Int. Paediatr. Dent..

